# The DDBJ Japanese Genotype-phenotype Archive for genetic and phenotypic human data

**DOI:** 10.1093/nar/gku1120

**Published:** 2014-12-03

**Authors:** Yuichi Kodama, Jun Mashima, Takehide Kosuge, Toshiaki Katayama, Takatomo Fujisawa, Eli Kaminuma, Osamu Ogasawara, Kousaku Okubo, Toshihisa Takagi, Yasukazu Nakamura

**Affiliations:** 1 DDBJ Center, National Institute of Genetics, Shizuoka 411-8540, Japan; 2 Database Center for Life Science, Chiba 277-0871, Japan; 3 National Bioscience Database Center, Japan Science and Technology Agency, Tokyo 102-8666, Japan

## Abstract

The DNA Data Bank of Japan Center (DDBJ Center; http://www.ddbj.nig.ac.jp) maintains and provides public archival, retrieval and analytical services for biological information. Since October 2013, DDBJ Center has operated the Japanese Genotype-phenotype Archive (JGA) in collaboration with our partner institute, the National Bioscience Database Center (NBDC) of the Japan Science and Technology Agency. DDBJ Center provides the JGA database system which securely stores genotype and phenotype data collected from individuals whose consent agreements authorize data release only for specific research use. NBDC has established guidelines and policies for sharing human-derived data and reviews data submission and usage requests from researchers. In addition to the JGA project, DDBJ Center develops Semantic Web technologies for data integration and sharing in collaboration with the Database Center for Life Science. This paper describes the overview of the JGA project, updates to the DDBJ databases, and services for data retrieval, analysis and integration.

## INTRODUCTION

Since 1987, the DNA Data Bank of Japan Center (DDBJ Center, http://www.ddbj.nig.ac.jp) at the National Institute of Genetics (NIG) has collected annotated nucleotide sequences in collaboration with GenBank at the National Center for Biotechnology Information (NCBI) and the EMBL-Bank at the European Bioinformatics Institute (EBI) within the framework of the International Nucleotide Sequence Database Collaboration (INSDC) (1). To cope with the recent surge in next-generation sequencing activity, DDBJ Center has launched new databases including the Sequence Read Archive (SRA) for raw and alignment data from next-generation sequencing platforms, the BioProject for sequencing project metadata and the BioSample for sample information in the framework of INSDC (2–4). These comprehensive resources for nucleic acid sequences and associated information comply with the uniform INSDC policy, which guarantees free and unrestricted access to the data archive (5).

A more recent demand is for an analysis platform for genotype and phenotype association using a large number of personal genomes. To exploit personal genomic data while respecting the privacy and informed consents of study participants, it is essential to establish a centralized repository for data management, and also a policy for data usage. In the United States and Europe, the database of genotypes and phenotypes (dbGaP) at NCBI (6,7) and the European Genome-phenome Archive (EGA) at EBI (8), respectively, serve as central repositories providing authorized access. As for policies, the National Institutes of Health (NIH) and the Wellcome Trust have established guidelines for sharing human subject data (9). This report focuses on the Japanese counterpart, the Japanese Genotype-phenotype Archive (JGA, http://trace.ddbj.nig.ac.jp/jga) in collaboration with our partner institute, the National Bioscience Database Center (NBDC, http://biosciencedbc.jp/en/) of the Japan Science and Technology Agency. NBDC has established guidelines and policies for sharing human-derived data, and the Data Access Committee (DAC) at NBDC reviews and makes decisions about data submission and usage requests from researchers.

JGA is intended to host information from several cohort studies in Japan, collecting genomic and medical records from Japanese individuals. In the Nagahama Zero-ji Prevention Cohort project (https://www.city.nagahama.shiga.jp/index.cfm/11,3709,96,558,html; information is available only in Japanese) conducted by the city and Kyoto University, genomic and other health-related information have been collected for over 10 000 inhabitants of Nagahama city, Shiga prefecture, Japan. The collected epidemiological data will be used to improve community health. Another study, Tohoku Medical Megabank Organization (http://www.megabank.tohoku.ac.jp/english) of Tohoku University, aims to develop a new medical system that combines medical and genomic information to support health and welfare in the northern (Tohoku) area of Japan, hit by the Great East Japan Earthquake. As part of this effort, the organization has completed whole-genome sequencing of 1000 healthy participants (http://www.megabank.tohoku.ac.jp/english/news/detail.php?id=826&c1=4) and collaborates with JGA for its metadata management.

In addition to our JGA activity, this paper also introduces the active collaboration with the Database Center for Life Science (DBCLS, http://dbcls.rois.ac.jp/en) to develop Semantic Web technologies for data integration and sharing. We list these achievements independently in the following sections. All resources described here are available from http://www.ddbj.nig.ac.jp.

## DDBJ ARCHIVAL DATABASES

### Database content

Between June 2013 and May 2014, the periodic release of the DDBJ annotated nucleotide sequence database increased by 7 329 558 sequence entries and 8 375 670 146 bp. The periodic release does not include whole-genome shotgun (WGS) and third party data (TPA) files (10). The DDBJ Center contributed 18.0% of the entries and 12.4% of the total base pairs added to the annotated nucleotide sequence data of INSDC. A detailed statistical breakdown of the number of records is available on our web page at http://www.ddbj.nig.ac.jp/breakdown_stats/prop_ent.html. In addition to the above data, DDBJ has released a total of 7 841 747 WGS entries, 253 011 CON entries, 748 TPA entries, 6374 TPA–WGS entries and 1272 TPA-CON entries as of 30 May 2014. In 2013, annotated sequences were submitted to DDBJ mostly from Asian countries: Japan (3540 times; 77.1%), Korea (223 times; 4.9%), India (223 times; 4.9%), China (143 times; 3.1%), Thailand (99 times; 2.2%) and other countries and regions (479 times; 10.4%).

Notable datasets released from the DDBJ sequence databases are listed in Table 1. These include genome assemblies of carnation, multiple strawberry species, a coral symbiont (*Symbiodinium minutum*), Japanese quail and Pacific bluefin tuna; genome survey sequences of radish, soybean and false killer whale; and transcriptomic sequences from a coral holobiont (*Porites australiensis*), a model plant (*Brachypodium distachyon*), and domesticated barley (*Hordeum vulgare* subsp. *vulgare*); and raw and aligned transcriptomic reads from a majority of mammalian primary cell types analyzed by the FANTOM5 consortium (11,12).

**Table 1.** List of notable data sets released from the DDBJ sequence databases from July 2012 to June 2013

Data type	Organism	Accession numbers of annotated sequences (number of entries)	Accession numbers of raw reads
Genome	Carnation (*Dianthus caryophyllus*)	WGS: BAUD01000001-BAUD01089083 (89 083 entries) scaffold CON: DF340864-DF357213 (16 350 entries)	DRR014087-DRR014092
	Strawberry (*Fragaria x ananassa*; assembled to eliminate heterozygous)	WGS: BATS01000001-BATS01220286 (220 286 entries) scaffold CON: DF266822-DF269452 (2631 entries)	DRR013866-DRR013871, DRR013873-DRR013882
	Strawberry (F. x ananassa; assembled with octoploidity)	WGS: BATT01000001-BATT01714282 (714 282 entries) scaffold CON: DF269453-DF338599 (69 147 entries)	DRR013873-DRR013882
	Strawberry (*Fragaria iinumae*)	WGS: BATU01000001-BATU01118549 (118 549 entries) scaffold CON: DF338600-DF339317 (718 entries)	DRR013884
	Strawberry (*Fragaria nipponica*)	WGS: BATV01000001-BATV01215530 (215 530 entries) scaffold CON: DF339318-DF339818 (501 entries)	DRR013885
	Strawberry (*Fragaria nubicola*)	WGS: BATW01000001-BATW01211274 (211 274 entries) scaffold CON: DF339819-DF340307 (489 entries)	DRR013883
	Strawberry (*Fragaria orientalis*)	WGS: BATX01000001-BATX01323675 (323 675 entries) scaffold CON: DF340308-DF340817 (510 entries)	DRR013886
	Coral symbiont (*S. minutum*)	WGS: BASF01000001-BASF01033816 (33 816 entries) scaffold CON: DF239013-DF260911 (21,899 entries)	DRR003834-DRR003864
	Japanese quail (*Coturnix japonica*)	WGS: BASJ01000001-BASJ01528405 (528 405 entries) scaffold CON: DF260915-DF266788 (5,874 entries)	DRR002288-DRR002301
	Pacific bluefin tuna (*Thunnus orientalis*)	WGS: BADN01000001-BADN01133062 (133 062 entries)	n/a
GSS	Radish (*Raphanus sativus* cv. Aokubi S-h)	GA872392-GA901611 (29 220 entries)	n/a
	Soybean (*Glycine max*)	LB000001-LB184894 (184 894 entries)	n/a
	False killer whale (*Pseudorca crassidens*)	DE737776-DE827431 (89 656 entries)	n/a
	coral symbiont (*S. minutum*)	GA393224-GA605429 (212 206 entries)	n/a
TSA	Coral (*P. australiensis*)	FX435232-FX505330, FX799345-FX804242 (74 997 entries)	DRR003753
HTC	*B. distachyon*	AK424275-AK440353 (16 079 entries)	n/a
EST	barley (*H. vulgare* subsp. *vulgare*)	5′ EST: DK584720-DK744249 (159 530 entries) 3′ EST: DK744250-DK887267 (143 018 entries)	n/a
	*B. distachyon*	5′-EST: HX789325-HX828682 (39 358 entries) 3′-EST: HX828683-HX867487 (39 247 entries)	n/a
Transcriptome	Majority of mammalian primary cell types sequenced by the FANTOM5 project	n/a	DRR008644-DRR010028, DRR013789-DRR013812

### Updates in the archival database services

Our web-based submission system (http://www.ddbj.nig.ac.jp/sub/websub-e.html) for annotated sequence submission has been implemented with a set of 11 templates designed for the most frequent types of sequence submissions (2). During 2014, the template system has been expanded with the release of new template sets for each category of sequence such as bacterial, plant and mammalian sequences. When using the templates, submitters provide nucleotide sequences with associated annotation using a spreadsheet-type editor with predefined mandatory and optional fields, a process that greatly reduces the overall complexity of the submissions process.

As an INSDC activity, DDBJ Center started, in February 2014, the BioSample database to organize sample information across archival databases. The DDBJ BioSample uses the same schema as NCBI's (13). In May 2014, the study and sample objects of the DDBJ Sequence Read Archive (DRA) had been migrated to the BioProject and BioSample records, respectively. Since this migration, registration in the BioProject and BioSample databases has been required before sequencing and alignment reads may be submitted to DRA. The DRA submission system has been improved to allow the submission of multiple experiment and run objects as a tab-delimited text file.

### Japanese Genotype-phenotype Archive

The JGA is a permanent archiving service for genotype and phenotype data of human individuals. JGA data are collected under a consent agreement that authorizes data release only for specific research use. The service is provided in collaboration with the National Bioscience Database Center (NBDC) of the Japan Science and Technology Agency. Data storage, management and distribution by JGA are governed by the NBDC policies and procedures for sharing human-derived data.

The DAC at NBDC reviews applications to submit data to JGA. The requests must fulfill the two conditions: (i) participant informed consent agreements for sharing and using data have been properly obtained; (ii) an entire study plan including data submission and use of public databases has been approved by an appropriate Institutional Review Board. Descriptions of these policies and guidelines can be found on the NBDC human database website at http://humandbs.biosciencedbc.jp. The English version of the websites will be prepared by the end of March 2015 for overseas researchers. Human data requiring authorized access should not be submitted to open-access INSDC databases such as SRA.

JGA accepts data that are de-identified by submitters. Acceptable data types include raw data formats from array-based or next-generation sequencing platforms and phenotype data associated with data samples. Processed or analyzed data such as alignments, assemblies and variations are also acceptable. Upon submission, the JGA team will archive the original data files in encrypted form in the database. Information in JGA is organized in a hierarchical JGA data model based on that of EGA (8). JGA assigns stable, unique identifiers prefixed by ‘JGA’ to studies and subsets of information from those studies, including samples, experiments, genotype data, analysis results and datasets for which consent has been given for use of the data.

The DAC also reviews requests from researchers to use JGA data sets for research. The DAC ensures that the stated research purposes are compatible with participant consent and that the Principal Investigator and institution will abide by the NBDC guideline and the specific terms and conditions imposed by a given dataset. Once access has been granted by DAC, datasets with access permission can be downloaded with secure downloading software.

It is the responsibility of users to establish a secured computing facility for local use of the downloaded data according to the NBDC security guideline. Approved applications are listed on the public NBDC page to preserve transparency of research conducted with JGA data. Approved users are required to submit annual reports to NBDC on data usage.

Information about available studies (summary level data) can be accessed freely on the JGA (https://ddbj.nig.ac.jp/jga/viewer/view/studies) and NBDC (http://humandbs.biosciencedbc.jp/data-use/all-researches-jp) websites. As of 22 October 2014, three studies are available at JGA: whole-exome sequencing study of 97 Japanese lung adenocarcinoma patients (JGA study accession number JGAS00000000001) (14), whole-genome sequencing study to detect expanded short tandem repeats associated with a brain disease spinocerebellar ataxia-31 (JGAS00000000002) (15) and whole-exome sequencing study of 23 initial low-grade gliomas and recurrent tumors resected from same patients (JGAS00000000004) (16).

## DDBJ SERVICE DEVELOPMENTS

### Updates in analytical services

DDBJ Center provides Web BLAST (17), ClustalW (18,19) and VecScreen (http://www.ncbi.nlm.nih.gov/tools/vecscreen/univec) services which receive requests from web interfaces. DDBJ Center also provides the new version of Web Application Programming Interface (API) for Bioinformatics (WABI) (20–22), a RESTful Web API service that can process requests from computer programs. The WABI service includes BLAST, VecScreen, ClustalW, MAFFT (23,24), a getentry system of data retrieval via accession numbers and an ARSA keyword search system for the DDBJ flat files (25).

These web applications and RESTful web services are hosted on the NIG supercomputer system (25). The current NIG supercomputer has been in operation since March 2012 (phase I) and the system was enhanced in March 2014 (phase II). The NIG supercomputer is a typical high-performance computing cluster system comprising calculation nodes for general purposes (504 thin-nodes each with 64 GB memory) and memory-intensive tasks including *de novo* assembly of sequencing reads (10 medium nodes each with 2 TB memory and 1 fat node with 10 TB memory). These nodes are interconnected with InfiniBand Quad Data Rate (QDR) (phase I) and Fourteen Data Rate (FDR) (phase II) by a complete bisection fat-tree topology. To allow the many calculation nodes to read and write the same files in parallel, the NIG supercomputer is equipped with 7 PB of the Lustre parallel distributed file system (http://www.lustre.org) for a high-performance large external storage system, and a 5.5 PB MAID for archiving the Sequence Read Archive data.

The DDBJ Read Annotation Pipeline (DDBJ Pipeline, http://p.ddbj.nig.ac.jp) is a high-throughput web annotation system for next-generation sequencing reads running on the NIG supercomputer (26). The pipeline comprises two components, the first for reference genome mapping and *de novo* assembly and the second for subsequent analysis such as structural and functional annotation with a Galaxy (27) interface. In 2014, two *de novo* assembly tools have been added. The first is Platanus, for highly heterozygous genomes (28), and the second is HGAP, for long reads derived from Pacific Biosciences sequencers (29). Users can perform contig annotations immediately after finishing the assembly process.

### Semantic representation of DDBJ data resources

To improve reusability of the sequence annotation data, we have developed the Resource Description Framework (RDF) version of DDBJ records in collaboration with DBCLS (30). The RDF data model has been made compatible with that of the Ensembl database based on the agreement at the first RDF summit held at DBCLS in May 2014 (https://github.com/dbcls/rdfsummit) so that users can query data and retrieve annotations at both sites in the same way. DDBJ continues to maintain its original semi-structured document format for which several bioinformatics tools and libraries have been developed (31–34), but a more efficient implementation is now possible using the RDF version, semantically compliant with the INSDC Feature Table Definition.

To semantically represent DDBJ nucleotide sequence annotation in RDF, we developed two ontologies: a DDBJ annotated nucleotide sequence ontology was manually prepared by defining classes and properties for describing entry metadata and feature qualifiers illustrating all the information in the existing DDBJ entries. The ontology explicitly specifies constraints between a given feature and possible qualifiers to maintain the quality of annotations by checking consistency with the INSDC specifications (2). The other ontology, DDBJ taxonomy, was automatically generated from the taxdump file of the NCBI Taxonomy database. There already exist several implementations of the taxonomic ontology, such as the one developed by the UniProt consortium (ftp://ftp.uniprot.org/pub/databases/uniprot/current_release/rdf/taxonomy.rdf.gz) or the OBO Foundry (http://purl.obolibrary.org/obo/ncbitaxon.owl). However, the former uses its own Uniform Resource Identifiers (URIs) and is not perfectly compatible with the NCBI Taxonomy, and the latter lacks many of the essential taxonomic annotations provided in the original NCBI Taxonomy. Because many different URIs for a same taxonomic identifier are already in use, we decided to primarily use Identifiers.org (35) URI as the resource URI (e.g. http://identifiers.org/taxonomy/9606) so that third-party developers can use our ontology without modification. Our two ontologies are available for browsing and downloading from our website (http://ddbj.nig.ac.jp/ontologies/).

## FUTURE DIRECTION

In Japan, most clinical data have been kept within a limited group of research collaborators. The centralized JGA system is expected to facilitate broader access and sharing of human data. DDBJ Center and NBDC equally collaborate with ongoing large-scale cohort and clinical studies to support efficient data sharing.

Another progress is our active collaboration with DBCLS. The web interface of DDBJ currently provides only links to search tools developed by DBCLS, but we host its developer team on the NIG campus and tighten the collaboration with this data-centric research center. On the backstage, DDBJ data are actively converted into the RDF-style with internationally acknowledged ontologies so that advanced queries using SPARQL Protocol and RDF Query Language become available.

The introduction of RDF bears significant implication in terms of smart integration with other omics information. NIG also stores information from National BioResource Project (NBRP) for collecting, preserving and sharing actual bioresources (http://www.nbrp.jp), and manages different types of resources such as phenotypes and metabotypes species-wise. By linking DDBJ with such information through the taxonomic classification, we can significantly improve the usefulness of genomic data from species-based genomics toward environmental and other interdisciplinary research area. Such amalgamation of research fields by information technology is our immediate goal and research collaboration is in progress.
